# Artificial Intelligence in Endoscopic Ultrasonography for Gastrointestinal Subepithelial Lesions: Evidence, Validation, and Clinical Translation

**DOI:** 10.3390/jcm15218440

**Published:** 2026-10-30

**Authors:** Elettra Merola, Leonardo Sosa-Valencia, Andrea Michielan, Nico Pagano, Maria Pina Dore, Claudio De Angelis, Abdenor Badaoui

**Affiliations:** 1 Department of Medicine, Surgery and Pharmacy, University of Sassari, 07100 Sassari, Italy; mpdore@uniss.it; 2 IHU-Strasbourg, Institute of Image-Guided Surgery, Université de Strasbourg, 67000 Strasbourg, France; leonardo.sosa-valencia@ihu-strasbourg.eu (L.S.-V.); abdenor.badaoui@chuuclnamur.uclouvain.be (A.B.); 3 Gastroenterology and Digestive Endoscopy Unit, Azienda Sanitaria Universitaria Integrata del Trentino (ASUIT), 38122 Trento, Italy; andrea.michielan@asuit.tn.it; 4 Unit of Gastroenterology, Ospedale Maggiore Della Carità di Novara, 28100 Novara, Italy; nicopagano@gmail.com; 5 Gastroenterology and Endoscopy Department, Koelliker Hospital, 10134 Torino, Italy; eusdeang@gmail.com; 6 Department of Gastroenterology and Hepatology, CHU UCL Namur, Université Catholique de Louvain, 5530 Yvoir, Belgium; 7 Institute for Translational Medicine and Liver Disease, ITM, UMRS_1110, Université de Strasbourg, 67000 Strasbourg, France; 8 Department of Gastroenterology and Hepatology, Hôpitaux Universitaires de Strasbourg, Université de Strasbourg, 67000 Strasbourg, France

**Keywords:** artificial intelligence, endoscopic ultrasonography, gastrointestinal stromal tumors, machine learning

## Abstract

Artificial intelligence is increasingly being applied to endoscopic ultrasonography for gastrointestinal subepithelial lesions, but the maturity of the evidence differs substantially across clinical tasks. This narrative review critically appraises contemporary studies of lesion detection and localization, wall-layer identification, diagnostic classification, pathological risk classification of gastrointestinal stromal tumors, human–artificial intelligence interaction, and potential management support. Thirty-one original studies published from 2021 through 2026 were included in the contemporary evidence map. Binary differentiation of gastrointestinal stromal tumors from leiomyomas or other selected comparators has the largest evidence base and includes multicenter, external, prospective, and real-time evaluations. Evidence for multiclass diagnosis, layer-of-origin assessment, small lesions, and pathological risk classification is less mature and is strongly influenced by case spectrum, reference standard, equipment, image selection, and validation design. No study has demonstrated that artificial intelligence improves tissue-acquisition strategy, surveillance, treatment selection, or patient outcomes in subepithelial lesions. Progress toward clinically useful decision support will require patient-level data separation, device- and center-held-out validation, analysis of complete examinations rather than curated images, explicit uncertainty handling, prospective human–artificial intelligence studies, and trials with endoscopy-specific management and safety outcomes.

## 1. Introduction

Gastrointestinal subepithelial lesions (SELs) encompass benign entities and neoplasms with malignant potential that may require tissue acquisition, surveillance, endoscopic resection, surgery, or multidisciplinary assessment. Endoscopic ultrasonography (EUS) is central to their evaluation because it defines lesion size, wall layer of origin, echogenicity, margins, and relationships with adjacent structures. Current European, American College of Gastroenterology, and American Gastroenterological Association guidance nevertheless emphasizes that EUS morphology alone cannot reliably distinguish all SEL subtypes, particularly solid hypoechoic lesions arising from the muscularis propria [1,2,3]. The challenge is especially relevant for small gastric SELs, for which a recent *Digestive Endoscopy* review highlighted the evolving roles of tissue acquisition, advanced EUS imaging, and artificial intelligence (AI) within an individualized diagnostic strategy [4]. Conventional EUS interpretation is also affected by interobserver variability [5], while tissue acquisition improves diagnostic confidence but is not invariably diagnostic [6].

AI has been studied as a way to reduce operator dependence and extract reproducible information from EUS images. Earlier investigations laid the foundations for contemporary EUS-based AI applications in gastrointestinal SELs. Initial studies used quantitative digital image analysis to distinguish GISTs from other mesenchymal tumors [7], followed by radiomics-based approaches to pathological GIST risk classification [8]. Subsequently, Minoda et al. demonstrated the feasibility of AI-assisted EUS diagnosis of GISTs in a pilot study published in 2020 [9]. These contributions provide historical context for the more recent studies included in the contemporary evidence map. Early meta-analyses, based on only seven to eight studies, reported pooled areas under the receiver-operating characteristic curve around 0.92–0.95 for AI-assisted diagnosis of gastrointestinal stromal tumors (GISTs) or SELs [10,11]. Since then, the field has expanded to multicenter datasets, external and temporal validation, prospective reader studies, real-time analysis, multimodal endoscopy-EUS systems, wall-layer classification, small-lesion assessment, and pathological GIST risk classification. A 2025 narrative review summarized recent EUS-based AI approaches for SEL diagnosis [12], while *Digestive Endoscopy* has recently reviewed AI applications in pancreatic EUS [13]. The unanswered question is therefore no longer whether an algorithm can classify selected EUS images, but which applications have evidence robust enough to assist an endosonographer during a real examination and which remain experimental.

**Figure 1:**
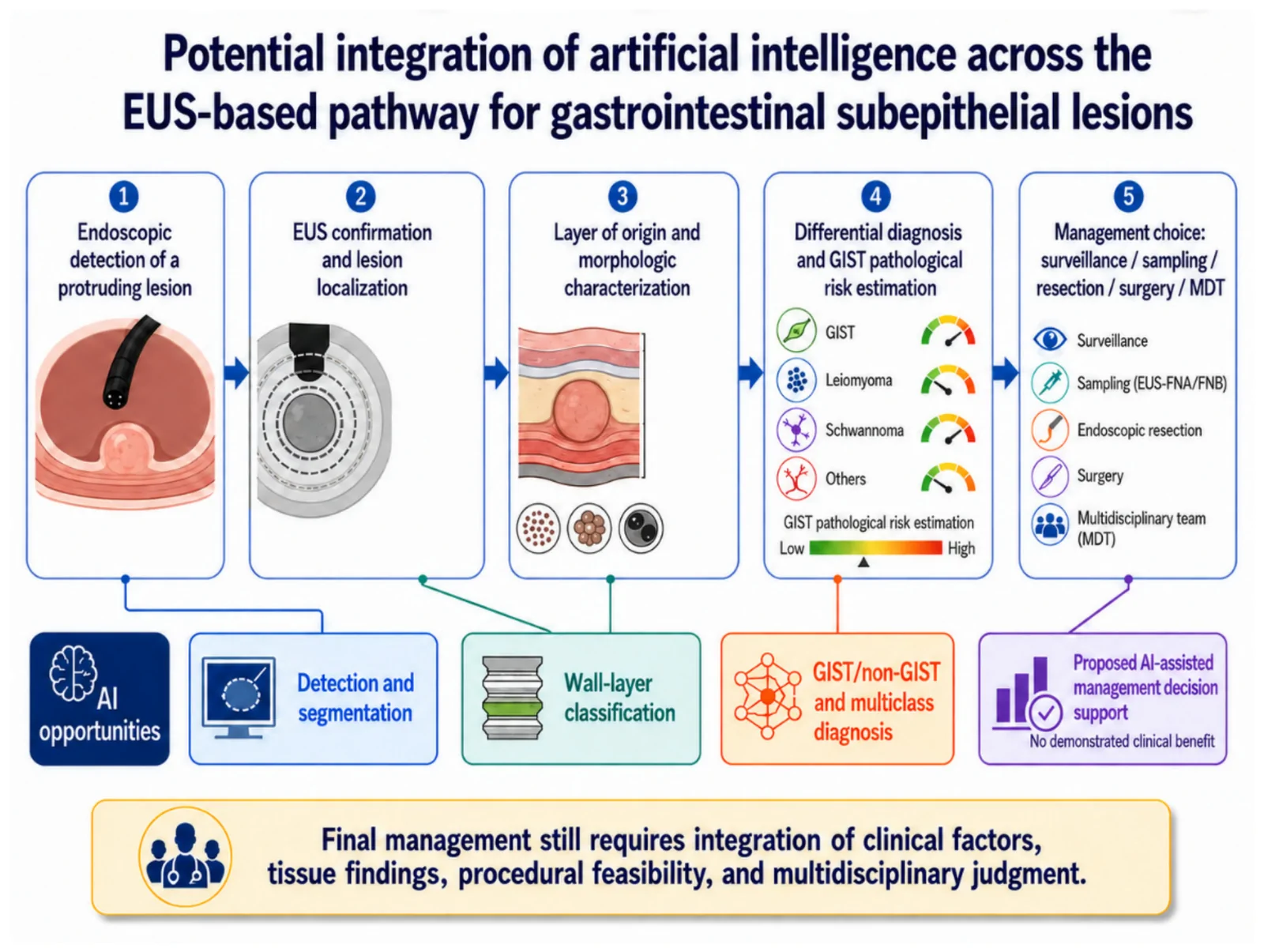
Artificial intelligence can support lesion localization, wall-layer identification, diagnostic classification, and gastrointestinal stromal tumor pathological risk estimation. Decisions regarding surveillance, tissue acquisition, endoscopic resection, or surgery still require integration of clinical context, tissue findings, procedural feasibility, multidisciplinary expertise, and patient preferences. The radial representation in panel 2 is a conceptual schematic and does not reproduce the conventional five-layer sonographic appearance of the gastrointestinal wall. AI-assisted management decision support is depicted as a proposed application without demonstrated clinical benefit for patients with gastrointestinal SELs. AI: artificial intelligence; EUS: endoscopic ultrasonography; GIST: gastrointestinal stromal tumor; MDT: multidisciplinary team.

This review focuses specifically on luminal gastrointestinal SELs. Its purpose is to compare the strength and limitations of evidence across clinical tasks, distinguish internal, temporal, pooled multicenter, external, and prospective real-time validation, separate pathological risk classification from true prognostic prediction, and define what would be required for EUS-based AI to progress from image interpretation to clinically meaningful decision support. A schematic overview of the potential points of AI integration across the EUS-based SEL pathway is shown in Figure 1.

## 2. Methods

This review was designed as a structured narrative synthesis rather than a systematic review. Accordingly, the literature search and study selection were intended to construct a contemporary, clinically oriented evidence map rather than to provide an exhaustive systematic enumeration of all eligible publications. The methodological limitations of this approach are discussed below.

An updated literature search was performed to identify original studies investigating the application of AI techniques to EUS for gastrointestinal SELs. The primary literature search was conducted in PubMed using (EUS OR “endoscopic ultrasonography”) AND (AI OR “machine learning” OR “deep learning”), with the PubMed five-year publication filter, and was last run on 31 July 2026; 391 records were retrieved. The five-year filter was selected to emphasize contemporary AI architectures and validation practices and was used as a discovery strategy rather than as a strict cutoff for the contextual literature. Reference lists and targeted searches using radiomics, texture analysis, computer-assisted diagnosis, artificial neural networks, and convolutional neural networks were used to identify studies not captured reliably by the broad query. Targeted update searches were performed through 9 September 2026. Earlier guidelines and non-AI EUS studies were retained only to provide clinical context.

Eligible original studies applied AI, machine learning, deep learning, radiomics, or quantitative computer-assisted analysis to EUS or microprobe-EUS data from gastrointestinal SELs and reported at least one anatomical, diagnostic, pathological-risk, human–AI, or management-related outcome. Studies limited to pancreatic, biliary, hepatic, or non-gastrointestinal EUS applications were excluded from the SEL evidence set. Reviews, meta-analyses, guidelines, and editorials were used for context but were not counted as original studies. Records identified through supplementary searches or reference tracing were manually deduplicated against the primary set by title, DOI, or PubMed identifier. Study eligibility assessment and selection were performed independently by two authors (E.M. and A.M.) within the structured narrative-review framework. Any discrepancies in study eligibility assessment were discussed between the two authors until consensus was reached. Methodological characteristics were extracted as reported in the original publications.

The primary PubMed search retrieved 391 records. Additional potentially relevant publications were identified through targeted searches and reference screening and were manually deduplicated against the primary search results. The final contemporary evidence map comprised 31 unique original studies published from 2021 through 2026: 24 studies addressing detection, anatomical characterization, or diagnosis (Supplementary Table S1) and nine addressing pathological GIST risk classification (Supplementary Table S2), with two studies contributing to both groups (24 + 9 − 2 = 31) (Supplementary Tables S1 and S2). Because the included studies addressed heterogeneous tasks, a single pooled risk-of-bias score was not considered appropriate. Instead, evidence was appraised descriptively according to patient-level data separation, reference standard, validation independence, input realism (selected still images versus video or real-time examination), diagnostic spectrum and device/center diversity, and whether a downstream clinical endpoint was evaluated. A task-based overview of the evidence and its principal limitations is provided in Table 1.

The validation terminology used throughout this review is defined in Table 2.

During manuscript preparation, ChatGPT (GPT-6; OpenAI, San Francisco, CA, USA) was used to assist with language editing, figure preparation and formatting. The authors critically reviewed and revised all AI-assisted output and take full responsibility for the scientific content and final text.

## 3. Current Evidence: Binary GIST Classification

Binary differentiation of GIST from leiomyoma or a limited non-GIST comparator is the most extensively studied EUS-AI task; detailed study characteristics are summarized in Supplementary Table S1. The earliest studies generally used retrospectively selected still images and internal or same-center validation. Object detection with patient-level voting, convolutional neural networks, handcrafted texture analysis, radiomics, and multimodal fusion all produced high internal performance in selected datasets [14,15,18,21,24,25,26]. A 2024 GoogLeNet study reported an overall accuracy of 95.15% for GIST-versus-leiomyoma classification using a random image-level training/test split (411/103 images) [22]. Fan et al. developed a Data Pyramid Structure for GIST classification and lesion-attribute prediction across five EUS centers, with multicenter ensemble-learning accuracy of 98.4% and an AUC of 0.927. However, their 70:30 training/test partition sampled data from each contributing center; this design does not constitute independent center-held-out external validation, and patient-level separation and complete histopathological verification of diagnostic labels were not clearly documented [23]. These designs are useful for model development but should not be interpreted as equivalent to independent clinical validation.

The stronger evidence comes from studies that evaluated models beyond retrospective image-level testing. Yang et al. developed a multicenter system and prospectively evaluated human–AI collaboration; among histologically confirmed cases, AI assistance increased endosonographer accuracy, although performance varied across probes and centers [16]. Lu et al. evaluated a model developed at two hospitals in patients from four other hospitals and reported more moderate lesion-level external performance, with sensitivity of 83.75%, specificity of 71.4%, and accuracy of 80.6% [17]. Dong et al. subsequently evaluated their model in a later multicenter cohort of 241 patients and in a separate prospective real-time cohort of 59 patients. Although the former was described as an external evaluation cohort, independent center-held-out validation was not clearly established. The model achieved an AUC of 0.95 and an accuracy of 91.7% in the later multicenter cohort, compared with an AUC of 0.86 and an accuracy of 86.5% in the prospective real-time cohort [20]. These estimates should be interpreted descriptively, since differences in cohort composition, sample size, and evaluation conditions prevent attribution of the observed performance differences specifically to real-time image acquisition. Nevertheless, the prospective findings support the feasibility of real-time implementation while emphasizing the need for independent validation under routine clinical conditions.

Multimodal systems provide another test of generalizability. Zhu et al. combined white-light endoscopy and EUS for three gastric lesion classes; performance for GIST classification was lower in the external hospital cohort than in temporal same-center validation, illustrating cross-center domain shift [32]. Other studies have combined clinical variables, radiomics, or structured EUS features [19,24,28]. Taken together, the binary GIST literature is the largest and includes the highest level of validation currently available in SELs, but reported accuracy is not directly comparable across studies because comparator lesions, equipment, reference standards, image selection, and validation designs differ.

## 4. Anatomical, Multiclass, and Adjunct EUS Tasks

AI may also support steps that precede or extend beyond binary histological classification (Supplementary Table S1). Three dedicated studies have addressed wall-layer assignment. SMT Master combined lesion and gastric-wall segmentation with layer classification and achieved approximately 81% external layer accuracy, but the external cohort contained only 26 patients [35]. A 2026 MedMamba model achieved 92.0% five-category accuracy with patient-level internal splitting; however, only 21% of reference labels were histopathological and lesion regions were manually selected [36]. In esophageal SELs, exact second-, third-, and fourth-layer classification was substantially harder than lesion detection: overall accuracy was 55.2%, increasing to 76.5% when the task was simplified to superficial versus fourth-layer origin [37]. This distinction matters for endoscopy because clinically aligned anatomical categories may be more useful than technically finer but less reproducible labels.

Multiclass diagnosis is supported by fewer studies. Hirai et al. classified five upper gastrointestinal SEL types with 86.1% accuracy in a 12-hospital dataset, but centers were pooled before development/testing rather than held out independently [31]. Zhu et al. evaluated GIST, leiomyoma, and ectopic pancreas using combined white-light endoscopy and EUS with separate temporal and external testing [32]. Li et al. reported high external and multicenter accuracy for a three-class multimodal system, whereas Chen et al. reported 84.9% external accuracy for four colorectal SEL classes and showed improved reader accuracy with AI assistance [33,34]. These studies broaden the lesion spectrum, but rare diagnoses remain underrepresented, and a model trained on a limited class set cannot be assumed to recognize an unrepresented lesion.

Small gastric SELs illustrate why study spectrum is as important as lesion size. High performance has been reported in restricted GIST-versus-comparator analyses, including lesions below 20 mm or 10 mm [18,20,27]. By contrast, Cai et al. prospectively evaluated a more heterogeneous set of small resected gastric SELs and reported an area under the curve of approximately 0.77 [19]. The contrast cautions against using a high estimate from a narrow binary task to justify surveillance or resection decisions in an unselected small-SEL population.

Adjunct EUS techniques and anatomical transportability remain much less studied. Tanaka et al. used contrast-enhanced harmonic EUS video tracking in 53 patients with GIST or leiomyoma and reported 90.6% accuracy, but the leave-one-out design did not provide independent external validation [29]. Elastography has been explored mainly for pathological GIST risk rather than broad lesion diagnosis [43]. Minoda et al. applied a gastric AI model to 52 esophageal, duodenal, and colonic lesions and reported high aggregate performance, but the site-specific groups were very small and highly imbalanced; specificity was 0% in the duodenal and colonic subgroups [30]. These studies support continued evaluation of dynamic and cross-site EUS information, but not direct transfer of gastric model performance to other anatomical locations.

## 5. Pathological GIST Risk Is Not Prognosis

Nine contemporary studies evaluated EUS-based classification of pathological GIST risk categories (Supplementary Table S2). The target definitions were not uniform. Some studies grouped very-low/low versus intermediate/high risk, Lu et al. classified very-low versus non-very-low risk in its binary model, and Cai et al. compared World Health Organization group 1 with group 4 [19,38,39,40,41,42,43,44]. Performance, therefore, should not be compared across studies as if they predicted the same endpoint.

The evidence also differs in validation strength. Liu et al. reported an area under the curve of 0.88 in a large 18-hospital dataset, but data from participating centers were partitioned across development and evaluation rather than tested in a fully center-held-out cohort [38]. Lu et al. performed geographic external validation; four-category classification was less accurate than the simplified binary task [40]. Several later single-center models reported high internal discrimination [24,41,42]. AI-assisted elastography yielded an area under the curve of 0.94 for a retrospectively derived stiffness cutoff, but the small-lesion subgroup included only four higher-risk tumors [43]. Hu et al. developed a four-category GIST pathological risk model based on the modified NIH criteria using 226 patients (504 EUS images), with external validation in 157 patients (176 images) from a separate hospital. The XGBoost model achieved an external AUC of 0.94 and accuracy of 80.68%. Interpretation remains limited by substantial risk-category imbalance, curated still images, manual region-of-interest annotation, and insufficiently explicit patient-level separation in the internal 8:2 data partition [44].

These studies estimate or reproduce pathological risk categories; they do not directly predict recurrence, metastasis, progression-free survival, or overall survival. No included study validated an EUS-AI risk output against longitudinal oncological outcomes. For endoscopic decision-making, this distinction is essential: a preoperative approximation of a postoperative pathological category is not yet a validated prognostic model.

## 6. Human–AI Interaction and Decision Support

For clinical endoscopy, the relevant comparator is often the endosonographer with versus without AI rather than AI versus a clinician in isolation. Reader studies have generally shown larger incremental gains among less experienced or less accurate readers, while benefit among experts is smaller or inconsistent [16,17,34] (Supplementary Table S1). Prospective and real-time evaluations demonstrate that model output can be delivered during an EUS examination [16,20], but they remain diagnostic studies.

For this review, clinical decision support is defined more narrowly as an AI output intended to influence a downstream action—such as tissue acquisition, surveillance, endoscopic resection, surgery, or multidisciplinary referral—and evaluated against the appropriateness, safety, yield, or clinical outcome of that action. By this definition, none of the included SEL studies has demonstrated clinical-impact decision support. A multimodal system mapped predicted diagnoses to predefined treatment categories, but this was a retrospective rule-based simulation rather than a prospective management study [33].

Human-factors evaluation is also incomplete. The available reader studies primarily measure diagnostic accuracy before and after AI assistance. No SEL study was designed to quantify automation bias by deliberately assessing whether an incorrect AI recommendation can overturn a correct human interpretation, nor has any study established when an operator should override or disregard a low-confidence output. Future reader trials should therefore report not only average accuracy but also discordant-case behavior, experience-dependent effects, confidence calibration, and the consequences of erroneous AI advice.

## 7. Tissue Acquisition and Management: The Missing Evidence

The most important evidence gap for an endoscopy audience is the absence of AI studies that directly improve EUS-guided tissue acquisition or subsequent management of SELs. The Asian Endoscopic Ultrasound Group consensus published in *Digestive Endoscopy* recommends tissue acquisition when histology would alter SEL management and favors fine-needle biopsy over conventional fine-needle aspiration when available [45]. Yet no included SEL-AI study prospectively selected which lesion should be sampled, identified the optimal target within a lesion, reduced needle passes, improved specimen adequacy or immunohistochemical yield, or guided needle choice.

There is adjacent evidence that AI can enter the EUS tissue-acquisition workflow. In pancreatic masses, Ashida et al. developed an AI-assisted real-time rapid on-site cytologic evaluation system and compared it with endosonographers and cytotechnologists [46]. This demonstrates technical feasibility in a different disease context, but it cannot be extrapolated to SELs, where histological architecture and immunohistochemistry are often central to diagnosis. For SELs, the most informative next trials would connect AI-assisted characterization to prespecified EUS-guided tissue-acquisition endpoints and then to downstream management.

Similarly, no study has shown that SEL-AI safely changes surveillance intervals, avoids unnecessary resection, selects endoscopic versus surgical treatment, reduces adverse events or costs, or improves oncological outcomes. These are the endpoints required to move from computer-assisted diagnosis to clinically meaningful endoscopic decision support.

## 8. EUS-Specific Barriers and Research Priorities

Several barriers are specific to EUS and should shape the next generation of studies (Table 3). First, EUS is an operator-dependent dynamic examination. Models trained on expert-selected still images do not encounter the same variation in probe position, compression, gain, depth, artifacts, and lesion visibility as a complete examination. Full-video or standardized sequence datasets, automated quality assessment, and prespecified lesion-level aggregation are therefore priorities. Second, the effective sample size is the number of independent patients or lesions, not the number of correlated frames; all images from one patient should remain within a single data partition before augmentation.

Third, equipment-related domain shift is particularly relevant because echoendoscopes, miniprobes, processors, frequencies, and image settings differ across centers. A multicenter dataset is not independent external validation when each center contributes data to both development and test sets. Device- and center-held-out evaluation should be reported explicitly. Fourth, diagnostic reference standards must match the task: resection pathology is preferable for pathological GIST risk, while expert EUS consensus is intrinsically weaker for wall-layer ground truth. Fifth, broad multiclass systems need deliberate representation of uncommon lesions and the ability to abstain on out-of-distribution inputs rather than forcing a familiar label.

Regulatory status, commercial availability, and access to representative datasets remain important barriers to clinical implementation. Although AI applications in EUS have progressed substantially, the systems evaluated in this review remain research-stage tools, with no reported regulatory authorization or routine commercial deployment specifically for gastrointestinal SEL assessment as of the final literature update in September 2026 [12,13]. This distinction is indication-specific and should not be generalized to EUS-AI systems developed for other clinical applications. Publicly accessible EUS datasets also remain scarce. An important recent initiative is CAD-SEL, a multicenter dataset released by Xu et al. in May 2026, comprising 4912 white-light endoscopy and EUS images from 641 patients with colorectal SELs, including 2095 EUS images from 421 patients [47]. However, its colorectal focus limits its representativeness for the broader spectrum of gastrointestinal SELs, particularly gastric GISTs. Larger, anatomically diverse, and consistently annotated public datasets are needed to facilitate independent benchmarking and external validation.

**Figure 2:**
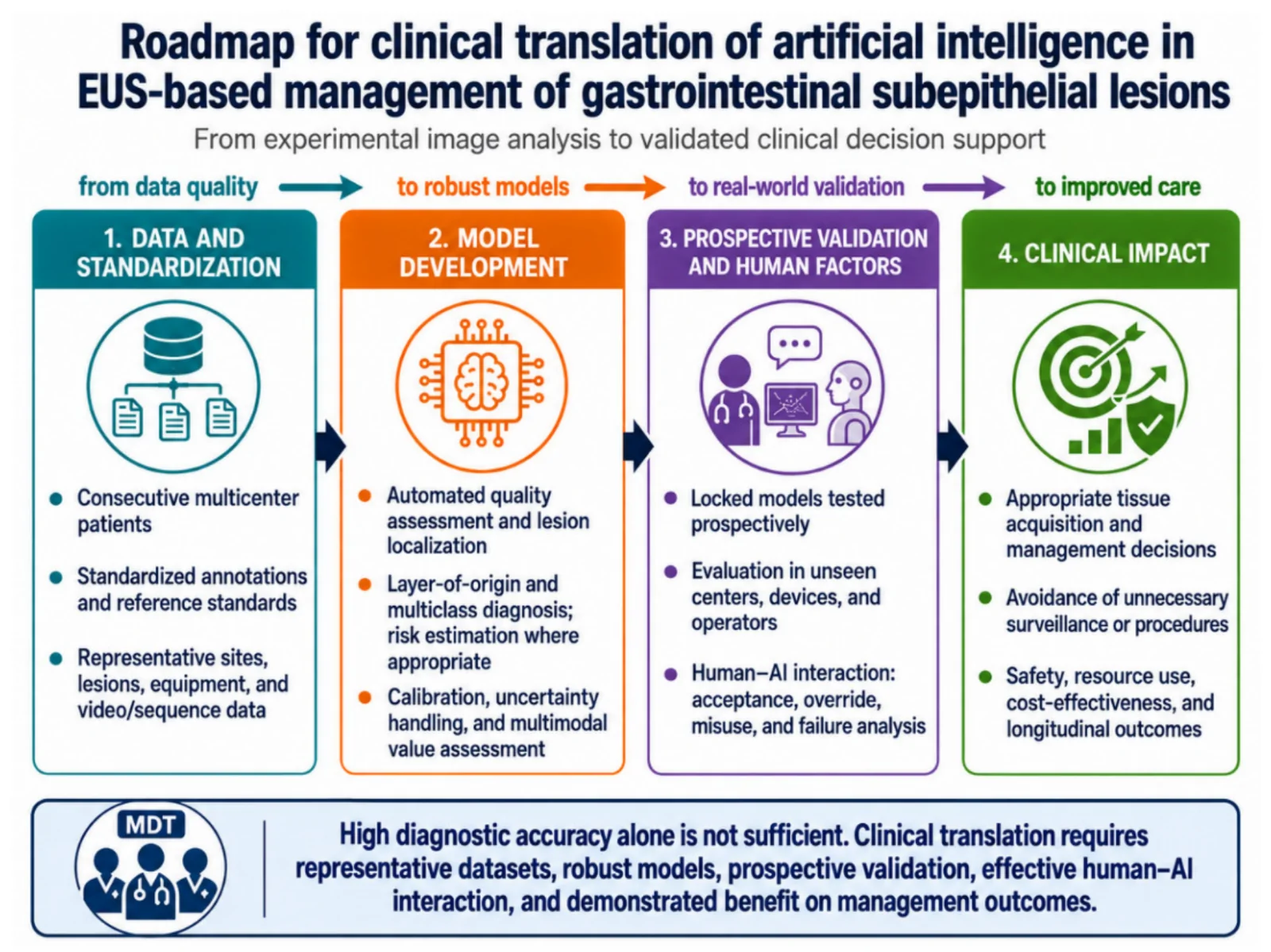
Four-stage roadmap for the clinical translation of artificial intelligence in EUS-based assessment of gastrointestinal subepithelial lesions, from representative datasets and clinically relevant models to independent prospective validation and demonstrated clinical impact. The final stage depicts proposed clinical-impact endpoints rather than benefits already demonstrated for patients with gastrointestinal SELs. Detailed research priorities and endpoints are summarized in Table 3. EUS: endoscopic ultrasonography; AI: artificial intelligence; MDT: multidisciplinary team.

Finally, prospective EUS-AI studies should be designed around endoscopy-specific clinical questions: whether AI changes the decision to sample, improves EUS-guided tissue acquisition, modifies surveillance or resection appropriately, and does so without automation-related harm. These priorities are more informative for translation than further optimization of internal image-level accuracy. These requirements are summarized in the clinical-translation roadmap shown in Figure 2.

## 9. Limitations of This Review

Several limitations of this review should be acknowledged. First, as a structured narrative review, the literature search was intended to provide a clinically oriented evidence map rather than an exhaustive systematic synthesis. Although the PubMed search was supplemented by targeted searches and reference screening, relevant studies may have been missed. Furthermore, a complete record of exclusions at each screening stage was not prospectively maintained, limiting the reproducibility of the selection accounting. Second, methodological appraisal was descriptive rather than based on a formal risk-of-bias assessment, and incomplete reporting of certain study characteristics limited the evaluation of methodological robustness. Finally, substantial heterogeneity in clinical tasks, lesion spectra, imaging inputs, reference standards, and validation strategies limits direct comparisons across studies. Publication and selective-reporting biases also cannot be excluded. Nevertheless, the task-based approach provides a clinically relevant synthesis of the available evidence and highlights the principal gaps that must be addressed before EUS-based AI can be translated into routine clinical practice.

## 10. Conclusions

EUS-based AI for gastrointestinal SELs has moved beyond proof-of-concept image classification, but the evidence is uneven across tasks. Binary GIST classification has the broadest and most externally tested evidence; layer-of-origin assessment, multiclass diagnosis, and pathological risk classification remain less mature, and pathological risk estimation should not be presented as validated prognosis. No study has yet demonstrated improved tissue acquisition, management, or patient outcomes. For endoscopy practice, the next step is not simply a higher internal area under the curve, but independent device- and center-held-out validation, prospective human–AI evaluation during complete EUS examinations, and trials that measure tissue-acquisition and management outcomes.

## Figures and Tables

**Table 1:** Evidence map for artificial intelligence applications in endoscopic ultrasonography assessment of gastrointestinal subepithelial lesions.

Clinical Task	Contemporary Evidence Base *	Strongest Validation Reported	What Current Evidence Supports	Main Limitation/Unresolved Question
Binary GIST classification	17 studies [14,15,16,17,18,19,20,21,22,23,24,25,26,27,28,29,30]	Independent external testing and prospective real-time evaluation	Diagnostic assistance in selected GIST-versus-comparator tasks	Restricted comparator spectra, curated images, device/center domain shift, and no demonstrated management impact
Multiclass SEL classification	4 studies [31,32,33,34]	External and multicenter testing in selected classes	Feasibility of broader classification in upper GI and colorectal SELs	Rare and unseen lesions remain underrepresented; class sets differ across studies
Layer-of-origin identification	3 dedicated studies [35,36,37]	Small external cohort, patient-level internal testing, and reader comparison	Anatomical assistance; clinically simplified categories may be more robust than exact adjacent-layer assignment	Reference standards are heterogeneous and often not pathological; real-time external validation is limited
Small gastric SEL assessment	4 diagnostic studies [18,19,20,27]	Prospective independent and external testing	Feasibility below 20 mm, including microlesions in selected tasks	Resection-selected populations and narrow binary tasks limit direct management inference
Pathological GIST risk classification	9 studies [19,24,38,39,40,41,42,43,44]	Geographic external validation in selected studies	Approximation of reported pathological risk groupings	Risk definitions differ; class imbalance is common; no longitudinal prognostic validation
Contrast-enhanced EUS/elastography	2 studies [29,43]	Retrospective internal evaluation	Proof-of-concept use of dynamic enhancement or stiffness information	Small cohorts, operator dependence, and insufficient independent multicenter validation
Human–AI/real-time evaluation	2 prospective/real-time studies plus several reader studies [16,17,20,34]	Prospective assistance and real-time diagnostic evaluation	AI can improve diagnostic classification for some readers	Automation bias, workflow effects, and downstream decisions have not been tested
AI-guided EUS tissue acquisition	0 SEL studies	None	No direct evidence	Need trials of sampling indication, target selection, adequacy, passes, diagnostic yield, and needle strategy
Management or patient outcomes	0 clinical-impact studies	None	Only retrospective rule-based management simulation has been reported [33]	Need surveillance, resection, safety, cost, and longitudinal outcome endpoints

* Categories overlap because individual studies may address more than one task. Counts refer to 31 unique original studies in the contemporary evidence map. Abbreviations: GIST: Gastrointestinal stromal tumor; SEL: Subepithelial lesion; GI: Gastrointestinal; EUS: Endoscopic ultrasonography; AI: Artificial intelligence.

**Table 2:** Validation terminology used in this review.

Term	Operational Definition	Interpretation
Internal validation	Random split, cross-validation, or held-out testing within the same source population or center(s) used for model development	Estimates performance within the development domain; does not establish transportability
Temporal same-center validation	Evaluation in a later patient cohort from the same center or institutional environment	Tests temporal drift but remains exposed to the same local acquisition and equipment ecosystem
Pooled multicenter testing	Data from several centers are mixed or jointly partitioned across development and test sets	Provides multicenter diversity but is not independent center-held-out external validation
External validation	Evaluation in patients from centers and/or equipment not represented during model development	Directly tests geographic or technical transportability
Prospective real-time evaluation	A locked model is applied prospectively during actual EUS examinations	Tests feasibility and diagnostic performance under clinical acquisition conditions; does not by itself prove management benefit
Clinical-impact validation	AI-assisted care is evaluated for downstream procedural, management, safety, resource, or patient outcomes	Required to establish clinical decision-support value beyond diagnostic discrimination

Abbreviations: EUS: Endoscopic ultrasonography; AI: Artificial intelligence.

**Table 3:** Endoscopic ultrasonography-specific priorities for clinical translation of artificial intelligence in subepithelial lesions.

EUS/SEL-Specific Problem	Recommended Study Design	Meaningful Endpoint
Expert-curated still images do not represent the full dynamic examination	Consecutive patients with complete videos or standardized image sequences; automated quality assessment and frame selection	Lesion-level performance during unselected examinations; failure rate on poor-quality frames
Multiple correlated frames inflate apparent sample size	Patient-level partitioning before augmentation; prespecified aggregation across views	Patient/lesion-level discrimination with confidence intervals
Echoendoscope, miniprobe, processor, frequency, and image-setting domain shift	Center- and device-held-out external validation with prespecified subgroup analyses	Calibration and performance across equipment and centers
Adjacent wall layers are difficult to distinguish reproducibly	Clinically aligned layer targets linked to procedural planning; explicit reference-standard hierarchy	Accuracy for management-relevant layer categories and effect on resection planning
Rare lesions and restricted class spectra	Broad pathology-rich cohorts with uncommon SELs and explicit out-of-distribution testing	Class-specific sensitivity/precision, calibration, and safe abstention
Uncertain indication and yield of EUS-guided tissue acquisition	Prospective AI-assisted EUS-TA studies linked to current FNB-based practice	Appropriate sampling, adequacy, diagnostic/immunohistochemical yield, needle passes, adverse events
Potential automation bias	Randomized or counterbalanced human–AI reader studies including deliberately discordant/incorrect AI outputs	Correct overrides, harmful reversals, confidence, time, and experience-dependent effects
Diagnostic accuracy is disconnected from management	Pragmatic impact trials integrating AI into surveillance/resection/tissue-acquisition pathways	Appropriate management, unnecessary procedures avoided, safety, cost-effectiveness, longitudinal outcomes

Abbreviations: EUS: Endoscopic ultrasonography; SEL: Subepithelial lesion; AI: Artificial intelligence; TA: Tissue acquisition; FNB: Fine-needle biopsy.

## Data Availability

No new data were created or analyzed in this narrative review. Data sharing is not applicable to this article.

## Supplementary Materials

The following supporting information can be downloaded at https://www.mdpi.com/article/10.3390/jcm15218440/s1, Table S1: Artificial intelligence and radiomics studies for detection, anatomical characterization, and diagnosis of gastrointestinal subepithelial lesions; Table S2: Artificial intelligence and machine-learning studies for pathological risk classification of gastrointestinal stromal tumors.

## Abbreviations

The following abbreviations are used in this manuscript:

SEL	Subepithelial lesion
EUS	Endoscopic ultrasonography
AI	Artificial intelligence
GIST	Gastrointestinal stromal tumor
FNA	Fine-needle aspiration
FNB	Fine-needle biopsy
MDT	Multidisciplinary team
GI	Gastrointestinal
SMT	Submucosal tumor
EUS-TA	Endoscopic ultrasonography-guided tissue acquisition
