# Supplementary material for: Artificial Intelligence in Endoscopic Ultrasonography for Gastrointestinal Subepithelial Lesions: Evidence, Validation, and Clinical Translation
Source: J Clin Med. 2026 Oct 30;15(21):8440. doi: 10.3390/jcm15218440 (PMC13764209; doi:10.3390/jcm15218440)
Supplement: Supplementary file 1 [file jcm-15-08440-s001.zip › jcm-4628941-supplementary.pdf]

Supplementary Materials

Table S1. Artificial intelligence and radiomics studies for detection, anatomical characterization, and diagnosis of gastrointestinal subepithelial lesions

Study	Design/setting	Site and clinical task	Cohort/data	Model/input	Input format	Reference standard	Patient-level split	Independent external validation	Validation	Key performance	Main limitation
Oh 2021 [14]	Retrospective, single center	Stomach; GIST vs leiomyoma with lesion detection	Training: 114 patients/376 images; test: 54 patients/170 images	CNN-based object detection; up to 5 representative EUS images per patient	Still images	Histopathology	Yes	No	Patient-level internal split; image- and patient-level analysis	Image-level AUC 0.92, accuracy 91.2%; patient-level voting AUC 0.99, accuracy 96.3%	Selected still images; no independent external validation
Hirai 2022 [31]	Retrospective, multicenter	Upper GI tract; five-class SEL diagnosis	631 cases, 16110 EUS images from 12 hospitals	Deep-learning multiclass image classifier	Still images	Predominantly pathological diagnosis, with selected clinical diagnoses	NR	No	Pooled multicenter development/test partition	Five-class accuracy 86.1%; for GIST vs non-GIST, sensitivity 98.8% and specificity 67.6%	Pooled multicenter split rather than center-held-out validation; uncommon classes sparsely represented
Minoda 2022 [30]	Retrospective, single center; transportability assessment	Esophagus, duodenum, and colon; GIST vs non-GIST using model developed in gastric lesions	Original gastric model: 173 lesions/2718 images; non-gastric test: 52 lesions (15 esophageal, 26 duodenal, 11 colonic)	Pre-existing CNN trained on gastric EUS images	Still images	Histopathology	NR	No	Testing in non-gastric anatomical sites	Overall AUC 0.98, accuracy 94.4%, sensitivity 100%, specificity 86.1%	Very small and highly imbalanced site-specific subgroups; only one non-GIST case in both duodenal and colonic groups
Seven 2022 [15]	Retrospective, single center	Upper GI tract; GIST vs leiomyoma	145 pathologically confirmed patients; 1362 EUS images; separate 45-patient validation cohort	CNN using conventional B-mode EUS images	Still images	Histopathology	Yes	No	Separate same-center validation cohort; comparison with expert EUS interpretation	Validation sensitivity 92.0%, specificity 64.3%, accuracy 87.0%; expert accuracy 63.0%	Single-center setting and relatively low specificity
Tanaka 2022 [29]	Retrospective, single center	Stomach; GIST vs leiomyoma using contrast-enhanced harmonic EUS	53 patients: 42 GISTs and 11 leiomyomas; 20-s CH-EUS video per patient, sampled into 200 frames	SiamMask tumor tracking and segmentation followed by ResNet-50 classification	Video frames	Surgical resection or EUS-guided tissue diagnosis	NR	No	Leave-one-out cross-validation; comparison with two expert endosonographers	Sensitivity 90.5%, specificity 90.9%, accuracy 90.6%; expert accuracy 88.7% (P = 0.68); tracking Dice coefficient 0.96	Small, imbalanced single-center cohort; no independent validation set
Yang 2022 [16]	Multicenter development with prospective human-AI evaluation	Upper GI tract; GIST vs leiomyoma	Retrospective development: 752 patients, 10439 images; prospective evaluation: 508 participants	ResNet-50 on EUS images	Still images	Histopathology where available	NR	NR	Multicenter testing across centers/devices; prospective reader-assistance study	In 132 histologically confirmed cases, AI assistance increased accuracy from 69.7% to 78.8%; among 80 GIST/leiomyoma cases, from 73.8% to 88.8%	Not all prospectively evaluated lesions had pathological confirmation; performance varied across probes and centers

Study	Design/setting	Site and clinical task	Cohort/data	Model/input	Input format	Reference standard	Patient-level split	Independent external validation	Validation	Key performance	Main limitation
Li 2023 [35]	Retrospective development with external and video testing	Upper GI tract; lesion segmentation, GI-wall segmentation, and originating-layer classification	Training: 201 patients/2721 images; internal validation 26 patients; external validation 26 patients; additional reader and video evaluations	Sequential deep-learning system for lesion segmentation, wall segmentation, and layer classification	Still + video	Expert EUS annotation with pathological confirmation where available	NR	Yes	Small external-center cohort plus video evaluation	Layer accuracy 84.4% internally, 80.7% externally, and 80.0% in video testing; lesion Dice 0.96/0.77 and wall Dice 0.82/0.74 in internal/external testing	Small external cohort; segmentation performance decreased externally; overlap among additional evaluation cohorts not always clear
Zhang 2023 [18]	Retrospective, multicenter data collection	Stomach; GIST vs leiomyoma/schwanno ma	485 cases, 3933 EUS images from 5 centers	1409 radiomic features; tumor-only model vs tumor plus adjacent muscularis propria	Still images	Histopathology	NR	No	Pooled random 7:3 development/test split	Combined tumor + muscularis propria model: AUC 0.95, accuracy 90.9% vs AUC 0.83, accuracy 76.8% for tumor-only model; lesions < 20 mm: AUC 0.96, accuracy 91.4%	Manual segmentation; centers pooled before splitting, therefore no center-level external validation
Lu 2023 [17]	Retrospective, multicenter	Stomach; muscularis-propria SELs, GIST vs non-GIST	Development: 367 patients/375 lesions/2057 images from 2 hospitals; external validation: 106 patients/108 lesions/914 images from 4 other hospitals	ResNeSt-50 on cropped B-mode EUS images	Still images	Histopathology from resection, surgery, or adequate tissue sampling	Yes	Yes	Geographic external validation; image- and tumor-level analysis; expert/novice comparison	External tumor-level sensitivity 83.75%, specificity 71.4%, PPV 89.3%, NPV 60.6%, accuracy 80.6%; image-level accuracy 78.1%	Manual ROI definition and image preprocessing; moderate specificity and NPV
Zhu 2023 [32]	Retrospective, two hospitals	Stomach; three-class GIST, gastric leiomyoma, and ectopic pancreas diagnosis using white-light endoscopy and EUS	1366 participants; 6406 white-light endoscopy images and 6314 images for combined-modality analysis reported	Multimodal multipath system with separate white-light endoscopy and EUS pathways plus hybrid integration	Still images	Histopathologica l diagnosis	Yes	Yes	Internal testing, temporal same-center validation (352 patients), and independent external-hospital validation	Internal AUCs: GIST 0.89, leiomyoma 0.89, ectopic pancreas 0.99; temporal AUCs 0.91/0.90/0.96; external AUCs 0.78/0.73/0.90, respectively	Retrospective, quality-screened images and manual lesion annotation; external performance decreased, especially for GIST and leiomyoma
Dong 2024 [20]	Multicenter diagnostic study with prospective real-time evaluation	Upper GI tract; GIST vs leiomyoma	Development cohort: 1,101 patients; later multicenter evaluation cohort: 241 patients; prospective real-time cohort: 59 patients	Deep-learning EUS diagnostic system	Still + real-time	Histopathology	Yes	NR	Later multicenter evaluation cohort (n = 241), described as external by the original authors, but independent center-held-out validation was not clearly established; separate prospective real-time evaluation (n = 59)	Later multicenter cohort: AUC 0.95, accuracy 91.7%; prospective real-time cohort: AUC 0.86, accuracy 86.5%	Independence of evaluation centers from development centers not clearly established; small prospective real-time cohort; no downstream management outcomes assessed

Study	Design/setting	Site and clinical task	Cohort/data	Model/input	Input format	Reference standard	Patient-level split	Independent external validation	Validation	Key performance	Main limitation
Fan 2024 [23]	Multicenter methodological study; five contributing EUS centers	Upper GI EUS; GIST vs non-GIST diagnosis and six lesion-attribute tasks with incomplete labels	Five-center EUS dataset; 614 GIST/non-GIST cases (181 GIST and 433 non-GIST) and 6,767 EUS images, calculated from the center-specific data reported in the original Table 2; number of unique patients not explicitly specified	Data Pyramid Structure (DPS), ResNet-18 backbone; unified ensemble (UELF) and federated-learning (UFLF) multicenter frameworks	Still images	GIST/non-GIST diagnostic labels reported; histopathological confirmation of all cases not explicitly documented. EUS attribute annotations derived from physician assessments	NR	No	Random within-center training/test split (70:30), preserving GIST/non-GIST proportions across all five centers; no independent center-held-out validation. Patient-level separation not explicitly reported	UELF (five-center analysis): accuracy 98.4%, sensitivity 98.9%, specificity 97.8%, AUC 0.927; DPS: accuracy 94.7%, AUC 0.930	No independent center-held-out validation; patient-level data separation and systematic histopathological confirmation of diagnostic labels not explicitly documented.
Cai 2024 [19]	Retrospective development followed by prospective independent testing	Stomach; SELs <2 cm, GIST vs non-GIST; pathological risk also evaluated	Development: 205 patients/5239 images; prospective testing: 178 patients/2404 images from 2 centers	Automatically optimized radiomics modeling system; EUS radiomics	Still images	Histopathology after endoscopic resection	Yes	NR	Prospective independent testing; comparison with 5 experienced endoscopists	Diagnostic AUC 0.76 in development and 0.77 prospectively; endoscopist AUCs 0.50-0.61	Restricted to resected small gastric lesions; representative-image selection and segmentation remained operator dependent
Liu 2024 [22]	Retrospective development with internal image-level testing and comparison with endoscopists	Upper digestive tract; GIST vs leiomyoma	514 EUS images; random image-level 4:1 split (411 training, 103 testing)	GoogLeNet-based convolutional neural network	Still images	Not specified	No	No	Random image-level internal split; comparison with endoscopists	Overall accuracy 95.15%; for GIST, sensitivity 94.4% and specificity 95.9%; for leiomyoma, sensitivity 95.9% and specificity 94.4%	Image-level internal testing in a restricted binary diagnostic task; no independent external validation
Lv 2024 [21]	Retrospective, single center	Stomach; GIST vs leiomyoma/schwanno ma	120 patients; ≥ 10 images initially available per lesion, with one high-quality still image selected for analysis	Manually defined ROI; GLCM, GLCM + global gray features, or GGCM features; SVM classifier	Still images	Postoperative histopathology	NR	No	Internal model evaluation; comparison with endoscopists	Best GGCM-SVM: AUC 0.90, accuracy 81.7%, sensitivity 81.4%, specificity 81.9%; endoscopist mean accuracy 69.3%	One manually selected image per lesion; manual ROI; no independent validation
Chen 2025 [34]	Multicenter study	Colorectum; four-class SEL diagnosis	510 histologically confirmed lesions from 7 hospitals	Multimodal white-light endoscopy + EUS AI system	Still images	Histopathology	Yes	Yes	Internal and external-center testing; human-AI reader evaluation	Four-class accuracy 92.1% internally and 84.9% externally; external WLE-only 69.4% and EUS-only 63.9%; AI assistance increased reader accuracy from 70.6% to 83.1%	Neuroendocrine tumors predominated; less common classes were relatively small
Duan 2025 [27]	Retrospective, single center with temporal testing	Stomach; small (< 20 mm) and micro (< 10 mm) GIST vs leiomyoma	574 EUS images; 2-6 images available per lesion	ResNet-50; predictions aggregated across images at lesion level	Still images	Histopathology	NR	No	Temporal same-center validation; image- and tumor-level analysis	Image-level AUC 0.84 for lesions < 20 mm and 0.832 for lesions < 10 mm; tumor-level aggregation increased AUC to 0.91 and 0.91, respectively	Same-center validation; selected multiple still images rather than complete examinations

Study	Design/setting	Site and clinical task	Cohort/data	Model/input	Input format	Reference standard	Patient-level split	Independent external validation	Validation	Key performance	Main limitation
Li 2025 [28]	Retrospective, multicenter	Upper GI tract; GIST vs non-GIST plus lesion-characteristic prediction	873 pathologically confirmed SELs, 9229 microprobe-EUS images from 5 hospitals	Interpretable deep-learning model using microprobe EUS; class activation maps and structured feature outputs	Still images	Histopathology for diagnosis; annotated EUS characteristics for lesion features	NR	Yes	Multicenter testing including 2 external datasets, one acquired with a different microprobe	Multicenter diagnostic accuracy 85.3%; accuracy for originating layer 88.3%, echogenicity 96.9%, and heterogeneity 77.3%	Performance varied across lesion characteristics and equipment; still-image based
Li 2025 [33]	Retrospective, multicenter comparative study	Upper GI tract; three-class GIST, neuroendocrine tumor, and leiomyoma classification plus lesion characterization	Development 523 lesions; external validation 88 lesions; multicenter test 274 lesions	Multimodal fusion of white-light endoscopy and microprobe EUS	Still images	Histopathology	Yes	Yes	Independent external validation and multicenter testing	Diagnostic accuracy 94.3% in external validation and 96.4% in multicenter testing; mean structured-feature accuracy 94.8%	Restricted diagnostic spectrum; management recommendations were simulated from predefined rules rather than tested clinically
Liu 2025 [24]	Retrospective, single center	Upper GI tract; GIST vs leiomyoma	265 patients: 141 GISTs and 124 leiomyomas; training 186 and internal validation 79	XGBoost fusion of baseline variables, conventional EUS morphology, and radiomic features	Still + data	Histopathology	Yes	No	Random patient-level 7:3 internal split; 10-fold cross-validation within training set	Multimodal fusion model: AUC 0.97, accuracy 93.1%, sensitivity 94.4%, specificity 91.7%	No independent external validation; reported image counts are internally inconsistent across sections of the article
Sun 2025 [25]	Retrospective, single center with temporal same-center testing	Upper GI tract (predominantly gastric; esophageal lesions also included); GIST vs leiomyoma	Development/validation: 681 patients; 13277 augmented grayscale images used for model development; independent temporal test: 22 patients (109 images)	Lightweight CNN with 7 convolutional units, approximately 1.43 million parameters	Still images	Histopathology	Yes	No	Five-fold cross-validation plus independent temporal same-center testing (2022-2024)	Cross-validation accuracy 96.2%, sensitivity 97.7%, specificity 94.7%, PPV 94.6%, NPV 97.7%; temporal test accuracy 94.5%, sensitivity 93.9%, specificity 95.4%	No independent-center validation; temporal test included only 22 patients; augmented image count greatly exceeds the number of independent patients
Zhang 2025 [37]	Retrospective, single center	Esophagus; SEL detection and originating-layer assignment	1445 patients, 1605 EUS images	YOLOv8s-seg for lesion localization/segmentation plus MobileNetV2 for layer classification	Still images	Predominantly expert EUS consensus, with pathological confirmation in a minority	Yes	No	Patient-level internal testing and reader comparison	Exact second-/third-/fourth-layer classification accuracy 55.2%; simplified superficial (second/third) vs fourth-layer classification accuracy 76.5%	Reference standard predominantly expert interpretation; exact adjacent-layer discrimination remained difficult; no external validation
Liu 2026 [26]	Retrospective, single center	Stomach; GIST vs other gastric tumors	219 patients, 4806 EUS images	Decision-level fusion of deep-learning, radiomic, and clinical models; t-test/LASSO feature selection	Still + data	Histopathology	NR	No	Internal train/test evaluation	AUC 0.95, sensitivity 100.0%, specificity 82.9%, PPV 60.0%, NPV 100.0%, accuracy 86.4%	Small internal test cohort with few GISTs; no independent external validation; manual image processing
Wang 2026 [36]	Retrospective, single center	Stomach; five-category wall-layer classification	320 patients screened; 305 patients and 1855 EUS images in final analysis	MedMamba state-space/deep-learning architecture	Still images	Histopathology in 21%; senior expert consensus plus stable follow-up in 79%	Yes	No	Patient-level 8:1:1 training/validation/test split; comparison with endoscopists	Overall accuracy 92.0% (95%CI 90.3%-93.7%), sensitivity 81.1% (95%CI 75.2%-87.0%), specificity 94.8% (95%CI 93.2%-96.4%)	Single-center internal validation; most reference labels were not pathologically confirmed; manual ROI cropping

Abbreviations: GIST: Gastrointestinal stromal tumor; CNN: Convolutional neural network; EUS: Endoscopic ultrasonography; AUC: Area under the receiver operating characteristic curve; GI: Gastrointestinal; SEL: Subepithelial lesion; CH-EUS: Contrast-enhanced harmonic endoscopic ultrasonography; AI: Artificial intelligence; PPV: Positive predictive value; NPV: Negative predictive value; ROI: Region of interest; DPS: Data Pyramid Structure; UELF: Unified Ensemble Learning Framework; UFLF: Unified Federated Learning Framework; GLCM: Gray-level co-occurrence matrix; GGCM: Gray-gradient co-occurrence matrix; SVM: Support vector machine; WLE: White-light endoscopy; LASSO: Least absolute shrinkage and selection operator; CI: Confidence interval. NR: Not reported or not sufficiently documented. Methodological coding: patient-level split = Yes only when separation by patient is documented; No for an explicitly image-level split without patient grouping; NR when not established. Independent external validation = Yes for testing at a held-out center or hospital site, No when absent, and NR when site independence cannot be determined. Input format describes the actual model input or evaluation mode; “still + data” indicates still images combined with structured variables.

Table S2. Artificial intelligence and machine-learning studies for pathological risk classification of gastrointestinal stromal tumors

Study	Design/setting	Prediction target and risk definition	Cohort/data	Model/input	Input format	Reference standard	Patient-level split	Independent external validation	Validation	Key performance	Main limitation
Liu 2021 [38]	Retrospective, multicenter; 18 hospitals	Binary lower- vs higher-risk GIST; very-low + low vs moderate + high risk	914 patients; 1824 EUS images	TN-USMA: intensity, size, and spatial-resolution normalization; ultrasound-specific self-supervised pretraining; deep image features integrated with tumor size, age, and sex through meta-attention	Still + data	Pathological risk classification	NR	No	Multicenter data partitioned into training/validation/test sets; robustness assessed across alternative multicenter partitions	AUC 0.881 (95%CI 0.82-0.92); accuracy 83.4% (95%CI 77.2%-88.5%); sensitivity 84.4% (95%CI 67.2%-94.7%); specificity 83.2% (95%CI 76.2%-88.8%)	Multicenter acquisition but no center-held-out external validation; manual tumor masks required; predicts pathological category rather than longitudinal outcome
Seven 2022 [39]	Retrospective, single center	Four-category AFIP risk; secondary binary low-risk (very-low + low) vs high-risk (intermediate + high)	Development: 55 patients/685 EUS images; validation: 15 patients/153 images	CNN with synthetic minority oversampling	Still images	Postoperative pathological risk classification	Yes	No	Separate later same-center validation cohort	Four-category validation: sensitivity 75%, specificity 73%, accuracy 66%; binary validation: sensitivity 99.7%, specificity 99.7%, accuracy 99.6%	Very small validation cohort; image-level prediction; striking binary estimates likely affected by small sample size and category collapsing; no external-center validation
Lu 2023 [40]	Retrospective, multicenter with external validation	Model 1: modified NIH four-category risk; Model 2: very-low vs non-very-low risk	Development/test: 1320 images; external validation: 656 images; development data from 2 hospitals and external data from 4 other hospitals	Two EUS-AI risk models	Still images	Modified NIH pathological classification	Yes	Yes	Geographic external validation; image- and tumor-level analyses	External four-category tumor-level: accuracy 74.5%, sensitivity 55.00%, specificity 79.0%, PPV 53.5%, NPV 81.6%; external binary very-low vs non-very-low tumor-level: accuracy 86.2%, sensitivity 94.4%, specificity 79.5%, PPV 79.1%, NPV 94.6%	Marked class imbalance; four-category sensitivity low, particularly for intermediate-risk lesions; binary task was very-low vs non-very-low, not lower- vs higher-risk
Cai 2024 [19]	Retrospective development with prospective independent testing at 2 centers	WHO group 1 vs group 4 among gastric GISTs < 2 cm	Development cohort: 94 GISTs of 205 SELs; prospective cohort: 84 GISTs of 178 SELs; group 4 represented 7 and 9 cases, respectively	Automatically optimized radiomics modeling system based on EUS images	Still images	Resection pathology and mitotic count	Yes	NR	Prospective independent testing	Development risk AUC approximately 0.73; prospective risk AUC 0.75	Restricted to resected gastric GISTs < 2 cm; very few group 4 tumors; manual representative-image selection and segmentation
Liang 2025 [41]	Retrospective, single center	Binary lower malignant potential (very-low + low) vs higher malignant potential (intermediate + high), modified NIH	318 gastric GIST patients; development n= 222, test n= 96, random 7:3 split	Structured preoperative clinical/EUS ML models; LASSO and logistic regression; predictors included EUS high-risk features, margin clarity, tumor diameter, and monocyte-to-lymphocyte ratio	Structured data	Postoperative modified NIH risk category	Yes	No	Internal random split; 10-fold cross-validation in training set	Logistic regression was best-performing classifier; AUC 0.91 in training and 0.92 in test set	Single-center internal validation; model uses structured clinical/EUS variables rather than raw EUS images; no independent external cohort

Study	Design/setting	Prediction target and risk definition	Cohort/data	Model/input	Input format	Reference standard	Patient-level split	Independent external validation	Validation	Key performance	Main limitation
Liu 2025 [24]	Retrospective, single center	Binary GIST risk classification; very-low + low vs intermediate + high according to modified NIH criteria	265 patients with GIST or leiomyoma; patient-level training n= 186 and validation n= 79; risk analysis restricted to GISTs	Baseline characteristics model, EUS morphology model, and multimodal fusion of clinical, EUS, and radiomic features using XGBoost; SHAP interpretation	Still + data	Complete pathology from surgical or endoscopic resection	Yes	No	Random 7:3 internal split; 10-fold cross-validation within training set; comparison with 3 experienced endoscopists	MMF risk AUC 0.99 (95%CI 0.98-0.99) in the main Results (0.992 in the abstract); accuracy 92.9%, sensitivity 78.9%, specificity 97.1%, PPV 78.4%, NPV 96.7%; endoscopist AUCs 0.73-0.76	No independent external validation; resection-selected population; image-count reporting and risk AUC are internally inconsistent across sections of the article
Qu 2025 [42]	Retrospective, single tertiary center	2008 revised NIH four-category risk; validation also evaluated binary very-low/low vs intermediate/high grouping	Development: 310 patients/1019 EUS images; subsequent same-center validation: 31 consecutive patients/96 images	TRPM based on conventional variables; DenseNet-121 DLRPM; combined CRPM	Still + data	Postoperative pathology and revised NIH risk classification	Yes	No	Subsequent 31-patient same-center validation cohort	Abstract: DLRPM accuracy 92.6%, AUC 0.93; CRPM accuracy 90.3%, AUC 0.91; TRPM accuracy 71.1%, AUC 0.84. Performance values are inconsistently reported in the Results section	Single-center study; only 31 patients in the subsequent test cohort; marked risk-category imbalance; internally inconsistent reporting of model performance
Zhang 2025 [43]	Retrospective, single center	Binary lower-risk malignancy (very-low + low) vs higher-risk malignancy (intermediate + high) using NIH classification	189 pathologically confirmed SMTs; 110 GISTs; 84 GISTs with available risk classification and EUS elastography	B-mode AI classification and segmentation followed by automated extraction of strain-elastography stiffness index (EUS-E-AI)	Still + elastography	Pathological NIH risk classification	NR	No	Internal retrospective evaluation; subgroup analysis in GISTs < 2 cm	EUS-E-AI cutoff 0.22: AUC 0.94 (95%CI 0.90-0.98), sensitivity 94.8%, specificity 90.7%, PPV 89.2%, NPV 95.4%, accuracy 92.6%; in GISTs < 2 cm AUC 0.842	Same-center derivation and testing of cutoff; strain elastography is operator-dependent; only 4 higher-risk tumors among lesions < 2 cm
Hu 2026 [44]	Retrospective multicenter study with external validation at a separate hospital site	Four-category modified NIH pathological GIST risk classification: very low, low, intermediate, high	Internal: 226 GIST patients/504 EUS images (two hospitals); external: 157 patients/176 EUS images (Yuzhong Hospital)	Physician-selected B-mode EUS images and expert-delineated ROIs; automated extraction of area, boundary irregularity, entropy, and grayscale SD; six ML classifiers evaluated	Still images	Post-resection histopathology with risk classification according to modified NIH criteria	NR	Yes	Random internal 8:2 training/test partition; ten-fold cross-validation with SMOTE restricted to training folds; external validation at a separate hospital site; patient-level grouping during internal splitting not clearly specified	XGBoost: internal AUC 0.97, accuracy 83.17%; external AUC 0.94, accuracy 80.68%	Marked class imbalance (only 15 intermediate- and 8 high-risk patients internally); selected still images and expert-defined ROIs; unclear patient-level grouping during internal splitting; no longitudinal prognostic validation

Risk-group definitions were retained as reported by the original studies and were not retrospectively harmonized. Binary endpoints were therefore not equivalent across studies: some combined very-low- and low-risk GISTs versus intermediate- and high-risk GISTs, whereas Lu et al. [40] classified very-low- versus non-very-low-risk tumors and Cai et al. [19] compared WHO group 1 with group 4. Reported performance should therefore not be directly compared across rows. Hu et al. [44] classified GISTs into four modified NIH pathological risk categories (very low, low, intermediate, and high), which were not harmonized with the binary endpoints reported by other studies.

Abbreviations: GIST: Gastrointestinal stromal tumor; EUS: Endoscopic ultrasonography; TN-USMA: Triple normalization-based model with ultrasound-specific pretraining and meta attention; AUC: Area under the receiver operating characteristic curve; CI: Confidence interval; AFIP: Armed Forces Institute of Pathology; CNN: Convolutional neural network; NIH: National Institutes of Health; EUS-AI: Endoscopic ultrasonography-based artificial intelligence; PPV: Positive predictive value; NPV: Negative predictive value; WHO: World Health Organization; SEL: Subepithelial lesion; ML: Machine learning; LASSO: Least absolute shrinkage and selection operator; SHAP: SHapley Additive exPlanations; MMF: Multimodal fusion model; TRPM: Traditional risk prediction model; DLRPM: Deep-learning risk prediction model; CRPM: Combined risk prediction model; SMT: Submucosal tumor; AI: Artificial intelligence; EUS-E-AI: Artificial intelligence-assisted endoscopic ultrasonography elastography; ROI: Region of interest; SD: Standard deviation; SMOTE: Synthetic Minority Oversampling Technique. NR: Not reported or not sufficiently documented. Methodological coding: patient-level split = Yes only when separation by patient is documented; No for an explicitly image-level split without patient grouping; NR when not established. Independent external validation = Yes for testing at a held-out center or hospital site, No when absent, and NR when site independence cannot be determined. Input format describes the actual model input or evaluation mode; “still + data” indicates still images combined with structured variables.
